# Iron Oxide Nanoparticles Carrying 5-Fluorouracil in Combination with Magnetic Hyperthermia Induce Thrombogenic Collagen Fibers, Cellular Stress, and Immune Responses in Heterotopic Human Colon Cancer in Mice

**DOI:** 10.3390/pharmaceutics13101625

**Published:** 2021-10-06

**Authors:** Mohammad Dabaghi, Seyed Mohammad Mahdi Rasa, Emilio Cirri, Alessandro Ori, Francesco Neri, Rainer Quaas, Ingrid Hilger

**Affiliations:** 1 Institute of Diagnostic and Interventional Radiology, Jena University Hospital, Friedrich Schiller University Jena, Am Klinikum 1, D-07740 Jena, Germany; mohammad.dabaghi@uni-jena.de; 2 Leibniz Institute on Aging Fritz Lipmann Institute (FLI), Beutenbergstraße 11, 07745 Jena, Germany; Mahdi.Rasa@leibniz-fli.de (S.M.M.R.); Emilio.Cirri@leibniz-fli.de (E.C.); Alessandro.Ori@leibniz-fli.de (A.O.); francesco.neri@leibniz-fli.de (F.N.); 3 Chemicell GmbH, Erseburgstrasse 22–23, 12103 Berlin, Germany; info@chemicell.com

**Keywords:** magnetic hyperthermia, thermal treatment, nanoparticles, colon cancer, DAMPs, thrombogenic collagen fibers, endocytosis signaling

## Abstract

In this study we looked for the main protein pathway regulators which were responsible for the therapeutic impact on colon cancers when combining magnetic hyperthermia with the chemotherapeutic agent 5-fluorouracil (5FU). To this end, chitosan-coated magnetic nanoparticles (MNP) functionalized with 5FU were intratumorally injected into subcutaneous human colon cancer xenografts (HT-29) in mice and exposed to an alternating magnetic field. A decreased tumor growth was found particularly for the combined thermo-chemotherapy vs. the corresponding monotherapies. By using computational analysis of the tumor proteome, we found upregulated functional pathway categories termed “cellular stress and injury”, “intracellular second messenger and nuclear receptor signaling”, “immune responses”, and “growth proliferation and development”. We predict TGF-beta, and other mediators, as important upstream regulators. In conclusion, our findings show that the combined thermo-chemotherapy induces thrombogenic collagen fibers which are able to impair tumor nutrient supply. Further on, we associate several responses to the recognition of damage associated molecular patterns (DAMPs) by phagocytic cells, which immigrate into the tumor area. The activation of some pathways associated with cell survival implies the necessity to conduct multiple therapy sessions in connection with a corresponding monitoring, which could possibly be performed on the base of the identified protein regulators.

## 1. Introduction

Along with the increasing number of cancers worldwide, colon cancer has always been one of the most common types. In 2020, more than 1.1 million new cases were estimated to be diagnosed [1]. Cost-effective and minimally invasive techniques such as colonoscopy and fecal immunohistochemistry have contributed to the early diagnosis of this cancer [2]. Additionally, the surgical removal of precursor lesions following early diagnosis, as well as improvements in adjuvant therapies, including radiotherapy and chemotherapy, have increased survival rates of this cancer type [3]. Despite significant progress in screening and treatment, colon cancer was estimated to cause more than 0.5 million deaths in 2020 [1].

In the last years, great emphasis has been devoted to the development of nanomedicines for the treatment of cancers. Hereto, the utilization of iron-oxide nanoparticles allows the exploitation of the magnetic core for heat-mediated cell inactivation purposes, termed “hyperthermia”. Hyperthermia is defined as treatment of tumor and tissues by temporarily raising the temperature to supra-physiological values. In this context, the temperature is typically increased up to 45 °C. The extent of tumor cell inactivation depends on the temperature and the duration of hating (thermal dose), which is expressed as the cumulative equivalent minutes at 43 °C (CEM43) [4,5]. It is widely accepted that the temperature of 43 °C for 60 min inactivates tumor cells. Furthermore, the nanoparticle’s coating is a versatile instrument for attaching specific chemotherapeutic drugs, particularly those inducing serious side effects or tumor resistance after repeated administration. Owing to the chemical versatility of nanoparticle coating, combinatorial treatment strategies using magnetic nanomaterials and chemotherapeutic agents (e.g., doxorubicin, methotrexate, camptothecin, etc.) have been studied in relation to different types of tumors (e.g., breast, bladder, colon cancer, etc.) [6,7,8,9,10]. Even though it was always shown that such combinatorial therapies led to a significant suppression of tumor growth in vivo and several proteins have been found to be responsible for such effects, there is still no comprehensive understanding of the responsible mechanisms and their interrelations, particularly in the view of the fact that the number of proteins produced by the 30,000–40,000 genes is three or four orders of magnitude higher [11]. Since most preclinical studies have been performed in a limited post-observation time, there have been no predictions on the biological activities and physiological roles of certain proteins, and finally there is no knowledge on how the contribution of each of the single therapy modalities (magnetic hyperthermia and chemotherapy) with respect to the combinatorial modality in terms of their impact on certain protein pathways. Since the highest impact of (magnetic) hyperthermia is obtained when heating and the chemotherapeutic drug are applied almost simultaneously at the tumor area [12], in the present study we assessed the combination of magnetically induced heating with the chemotherapeutic agent 5FU. 5FU has long been used to treat colon cancer [13]; commonly before and after colorectal surgery or as part of the treatment of metastatic disease. 5FU disrupts both DNA and RNA synthesis and interferes with thymidylate synthase, resulting in DNA breaks and cell death [14]. Hyperthermia can enhance the cytotoxicity of 5FU by interfering with the DNA repair process and denaturing DNA polymerases [15].

To this end, we functionalize chitosan-coated magnetic nanoparticles (MNP) with 5-fluorouracil (5FU), inject them intratumorally into a subcutaneous human colon cancer xenograft in mice, and expose the tumor bearing tissue to an alternating magnetic field. We termed the combination of magnetic hyperthermia and chemotherapy as “thermo-chemotherapy”. In particular, we wanted to unveil the main protein pathway regulators, which were responsible for the therapeutic impact of the combined thermo-chemotherapy, by analyzing its effects in comparison to the respective single modalities. We found a distinct impact on the tumor extracellular matrix, the induction of thrombogenic collagen fibers, a distinct impact on second messenger, and nuclear receptor signaling as a response to cellular stress in relation to the combined thermo-chemotherapy. We further associate the presence of acute phase response signaling and caveolar-mediated endocytosis signaling with the recognition of damage associated molecular patterns (DAMPs) and the presence phagocytic cells which immigrated into the tumor area. The activation of pathways associated with cellular growth are related with the presence of surviving cells, which elucidates the need to conduct multiple therapy cycles, potentially under the surveillance of a dedicated marker-based monitoring.

## 2. Materials and Methods

### 2.1. Magnetic Nanoparticles Functionalized with 5-Fluorouracil

The core of the superparamagnetic iron oxide nanoparticles (MNPs) were synthetized by co-precipitation, i.e., by the addition of an alkaline solution to an aqueous solution of Fe^3+^ and Fe^2+^ chloride. Afterwards, a thermic reaction to Fe^2+/3+^-oxide was undertaken. Iron oxide crystals were purified with several washing steps. The iron oxide crystals were separated magnetically after each step. Nanoparticle coating with chitosan was performed by chemisorption and excess of chitosan was removed by several washing steps. 5-fluorouracil (5FU) powder was obtained from Sigma-Aldrich (Sigma-Aldrich Chemie GmbH, Steinheim, Germany). 5FU was electrostatically incorporated into the chitosan MNP coating via inter-molecular hydrogen bonding interactions between hydroxide (OH) in chitosan molecule and oxygen (O) in 5FU molecule as follows: 500 µL of 5FU diluted in 1 N NH_4_OH solution at a concentration of 10 mg/mL was added to 500 µL MNP-suspension (1 mg Fe/mL). The mixture was gently shaken for 20 min at room temperature. Subsequently, the MNPs were separated magnetically and re-suspended in distilled water. The concentration of coupled 5FU was measured via a UV-vis spectrometer (λ = 265 nm; Ultrospec 4300 pro UV/Visible Spectrophotometer, Amersham Pharmacia Biotech, Freiburg, Germany). Hereto, we used a calibration curve plotting the absorption (λ = 265 nm) against the concentration of 5FU in mg/mL to measure the given concentration of 5FU and the concentration of 5FU in the supernatant after washing. For intratumoral injection of free 5FU, a 5FU injection solution (Accord Healthcare GmbH, Munich, Germany) was used. The size, ζ-potential, and polydispersity index (PdI) of the MNPs was determined via dynamic light scattering (solvent: distilled water, DLS, Zetasizer Nano ZS; Malvern Instruments GmbH, Herrenberg, Germany), and the MNP’s iron was quantified via AAS [16]. The heating potential of the MNPs was determined as described previously [17].

### 2.2. Animals and Ethics

To assess the protein profile of tumor after magnetic hyperthermia, 8 weeks old female athymic nude mice (Hsd: Athymic Nude-Foxn1^nu^, Envigo RMS GmbH, Venray, The Netherlands) were used in this study. All experiments were approved by the regional animal care committee (Reg.-Nr. 02-058/16; Thüringer Landesamt für Verbraucherschutz, Bad Langensalza). Tumor induction was performed by subcutaneous injection of human colorectal cancer HT29 cells suspended in Matrigel™ (Becton, Dickinson and Company, Franklin Lakes, NJ, USA). Experiments were started when tumors had reached a volume of 100 to 250 mm^3^.

In this study, four (4) independent animal groups were defined as follows: group C: the combinatorial treatment group (with 5FU-MNP injected intratumorally, i.e., 5FU-MNP: 0.25 mg Fe per 100 mm^3^ tumor tissue, and tumors exposed to an alternating magnetic field, *n* = 11); group H: the magnetic hyperthermia treatment alone group (with intratumorally injected MNP (same iron dose as group C exposed to an alternating magnetic field, *n* = 11) to monitor the contribution of magnetic hyperthermia in the combinatorial treatment; group F: the 5FU based chemotherapy alone group (with intratumorally injected free 5FU (without MNP), same dose as group C, without the exposure to an alternating magnetic field, *n* = 10) to study the impact of 5FU when coupled with MNP in the combinatorial treatment; and group N: the untreated group (no MNP, no 5FU, no exposure to an alternating magnetic field, *n* = 11) to monitor the overall therapeutic outcome of the treatments.

Two post observation time points after magnetic hyperthermia (day 0) were used to assess the dynamics of the therapeutic impact: (S) short-term (until day 1 after the last tumor therapy) and (L) long-term observation time (14 days after the last tumor therapy). The tumor volume immediately before treatment was used as reference for the changes of tumor volume during the post-treatment observation period, and data were normalized to this reference value (relative tumor volumes). Animals were sacrificed on day 1 and day 14 post last tumor therapy (early (S) and late (L) post-observation periods, respectively) and tumor tissue was excised for further analysis immediately thereafter.

### 2.3. Magnetic Hyperthermia

Magnetic nanoparticles (5FU-MNPs or MNPs) were intratumorally injected. On the following day as well as 7 days later, magnetic hyperthermia was performed by exposure of tumors to an alternating magnetic field (AMF; *H* = 15.4 kA/m, *f* = 435 kHz) for 60 min. During the magnetic hyperthermia treatment, the body temperature (rectal) of the animals and the temperature of the tumor surface were monitored by utilization of optical fiber temperature sensors as well as an infrared thermographic camera (NEC Avio Infrared Technologies Co. Ltd., Tokyo, Japan). In particular, the total temperature dose (CEM43T90) of group C was of 23 +/− 22 min for the magnetic hyperthermia treatment. In group H, the total CEM43T90 was 17 +/− 10 min. The CEM43T90 represents the Cumulative Equivalent Minutes of a tumor area of 90% at 43 °C (CEM43T90). This definition is based on a mathematical description of the exponential relationship between temperature and exposure time [4].

### 2.4. Tumor Sample Extraction for Proteomics Analysis

At given post-observation times (see above), animals were killed and tumors were isolated. Randomly selected tumor tissues samples (equal weight amount), one tumor per animal, *n* = 4 to 5 animals per group) were provided with RIPA lysis buffer in presence of protease and phosphatase inhibitors (Roche Diagnostics GmbH, Mannheim, Germany) and homogenized using the gentleMACS-Octo Dissociator (Miltenyi Biotec B.V. & Co. KG, Bergisch-Gladbach, Germany). Subsequently, proteins were separated by centrifugation at 15,680 × *g* for 5 min and 3 samples per animal group were taken for further analyses.

### 2.5. Sample Preparation for Proteomics Analysis

For proteomics analysis, samples were prepared according to Buczak et al. (2020) [18] Briefly, samples were sonicated (Bioruptor Plus, Diagenode, Belgium), boiled at 95 °C for 5 min, reduced and alkylated with iodoacetamide (IAA, final concentration 15 mM) for 30 min at room temperature in the dark. Proteins were precipitated overnight at −20 °C after adding ice-cold acetone. The following day, samples were centrifuged and the supernatant was carefully removed. Pellets were washed with ice-cold 80% (*v*/*v*) acetone in water, then centrifuged and air-dried. After adding 25 µL of digestion buffer (1 M Guanidine, 100 mM HEPES, pH 8, Sigma-Aldrich Chemie GmbH, Steinheim, Germany), proteins were digested first with LysC (Wako, Neuss, Germany) (1:100 (*w*/*w*) enzyme:protein ratio) for 4 h at 37 °C under shaking and then with trypsin (Promega, Walldorf, Germany) (1:100 (*w*/*w*) enzyme:protein ratio) overnight at 37 °C under shaking. The day after, digests were acidified with TFA (final concentration of 10% (*v*/*v*)), heated at 37 °C and then desalted with Waters Oasis^®^ HLB µElution Plate 30 µm (Waters Corporation, Milford, MA, USA) under a soft vacuum following the manufacturer instruction. Dried samples were stored at −20°C until analysis.

### 2.6. LC-MS Data Dependent (DDA) and Independent Analysis (DIA)

Prior to analysis, samples were reconstituted in in MS Buffer (5% acetonitrile, 95% Milli-Q water, with 0.1% formic acid) and spiked with iRT peptides (Biognosys, Schlieren, Switzerland). An amount of 1 μg peptides were separated in trap/elute mode using the nanoAcquity MClass Ultra-High Performance Liquid Chromatography system (Waters, Waters Corporation, Milford, MA, USA) with a flow of 0.3 μL/min over a C18 trap and a C18 analytical column. Solvent A was water and 0.1% formic acid, solvent B was acetonitrile and 0.1% formic acid. A nonlinear gradient of solvent B (from 0 to 40%) was applied for 120 min. Total run time was 145 min including equilibration and conditioning. The LC was coupled to an Orbitrap Exploris 480 (Thermo Fisher Scientific, Bremen, Germany) using the Proxeon nanospray source heated at 300 °C, with a spray voltage of 2.2 kV. Ion funnel radio frequency was set to 30%.

For DDA data acquisition, full scan MS spectra were acquired with resolution of 60,000 FWHM, maximum filling time of 20 ms and AGC target of 3 × 106 ions. Precursor ions were selected for fragmentation with a Top 20 method (normalized collision energy of 27%, quadrupole isolation (1.6 *m*/*z*), resolution 15,000 FWHM, only 2+ −6+ precursor ions, no isotopes). For DIA data acquisition, full scan mass spectrometry (MS) spectra were acquired in profile mode with resolution of 120,000 FWHM, maximum filling time of 60 ms and AGC target of 3 × 106 ions. Mass range for DIA scan acquisition was divided into 40 mass window segments of differing widths. MS/MS spectra were acquired with a resolution of 30,000 FWHM and a stepped normalized collision energy higher collisional dissociation fragmentation (25, 27.5, and 30%). For data acquisition and processing of the raw data Xcalibur 4.3 (Thermo Scientific, Bremen, Germany) and Tune version 2.0 were used.

### 2.7. Data Processing

Acquired data were processed using Spectronaut Professional v13.10 (Biognosys, Schlieren, Switzerland). For library creation, the DDA and DIA raw files were searched with Pulsar (Biognosys, Schlieren, Switzerland) against the mouse UniProt database (Homo sapiens, entry only, release 2016_01) with a list of common contaminants appended, using default settings. For library generation, default BGS factory settings were used. DIA data were searched against this spectral library using BGS factory settings, except: Proteotypicity Filter = Only Protein Group Specific; Major Group Quantity = Median peptide quantity; Major Group Top N = OFF; Minor Group Quantity = Median precursor quantity; Minor Group Top N = OFF; Data Filtering = *q*-value sparse; Normalization Strategy = Local normalization; Row Selection = Automatic. Relative quantification was performed in Spectronaut for each paired comparison using the replicate samples from each condition. The data (candidate table) and data reports (protein quantities) were then exported and further data analyses and visualization were performed with Rstudio using in-house pipelines and scripts. To select significant proteins, a log_2_ fold change cutoff of 0.58 and a *q*-value < 0.05 were defined.

### 2.8. Statistical Models for Proteomics

Statistical models for analyzing the proteome data followed the study design, which consisted of one time point each (short-term or long-term observation time, see above) and three factors (combined treatment, magnetic hyperthermia alone, and intratumoral 5FU treatment alone). To determine the specific signature of each experimental therapy arm, we compared the effects obtained from pairwise comparisons (combined therapy vs. non-treated control) for one specific post-treatment observation time. For protein ontology and up-stream regulator analysis, ingenuity pathway analysis (IPA, v62089861) has been used. The proteins with *q*-value < 0.05 and absolute number of log_2_ fold change higher or equal to 0.58 were uploaded in the IPA software and analysis was performed using default parameters. The functional category of each pathway was also extracted from IPA. The plotting the results was performed using R programing (v3.6.0) with the following packages: ggplot2 (v3.2.1), pheatmap (v1.0.12) and VennDiagram (v1.6.20). In all cased *p*-values were adjusted with particular consideration of the high-dimensional aspect in such analyses.

## 3. Results

### 3.1. Features of the Nanoparticles Carrying 5FU

In general, the nanoparticle core (mean diameter between 8 and 14 nm) was composed of a several magnetite/maghemite clusters (Fe3O4/γ-Fe_2_O_3_), which have been embedded in a dextran matrix. Such cores were additionally coated with chitosan, which carried electrostatically attached 5FU molecules (5FU-MNPs). Our MNPs exhibited the following morphological features: The mean hydrodynamic diameter was 176 ± 7 nm, the PdI of 0.17 ± 0.01, and the ζ-potential of −27.9 ± 0.5 mV. The iron content of our MNPs was 1.1 ± 0.2 mg Fe/mL of MNP suspension medium and the heating potential equaled 462 ± 70 W/g Fe. UV-spectroscopic analysis revealed that the concentration of coupled 5FU equaled 138 ± 14 µg/mg Fe. With these features our 5FU-MNPs were qualified for their utilization in the combined thermo-chemotherapeutic treatment.

### 3.2. Impact on Tumor Volumes

The determination of tumor volumes showed that the thermo-chemotherapeutic tumor treatment via the utilization of our 5FU-MNPs in combination with magnetic hyperthermia (group C, see methods) resulted in a distinct tumor regression, which was much more evident than that of magnetic hyperthermia alone, whereas the intratumoral application of 5FU (comparable concentration as intratumorally applied in group C, see methods) had almost no effect. In other words, the thermo-chemotherapeutic tumor treatment has been considerably more effective than magnetic hyperthermia or 5FU only, Table 1).

### 3.3. Global Impact of Therapy on the Tumor Tissue Proteome

In order to shed more light into the impact of the mentioned therapeutic arms on tumor development, which was macroscopically seen by measuring tumor volumes, we performed a series of proteome analysis. In this context, the global analysis showed that more than 5000 protein groups could be identified in each of the three investigated therapeutic arms (data not shown). Additionally, principal component analysis (PCA) revealed a low intra-group variance of protein expression between the replicates in each group compared to the inter-group differences (Supplementary Figure S1). In detail, the PC1 (Dim, the maximum variance of the data, the most important data variance direction) indicates that the largest variation is between the combination treatment and the other treatment groups (22% of the total variance between our tumor tissue samples). Further on, PC2 (Dim2, the second most important variance direction, it is orthogonal to the PC1 axis) mainly reveals the differences between the short-term and long-term treatments; it explains 15.8% of the total variance. Interestingly, the treatment of tumor with hyperthermia and 5FU (H-L and F-L in Supplementary Figure S1) as monotherapy are close to the control groups in the PCA plot, which explains a comparably low long-term impact of the latter mentioned ones compared to the thermo-chemotherapy approach.

Volcano plots of tumor protein expression (in comparison to non-treated controls) showed that the amplitude (*x*-axis) and the statistical significance of differential protein expression were almost similar between the combined thermo-chemotherapy vs. the monotherapies in the short-term observation time. Nevertheless, the impact of the combined thermo-chemotherapy and that of hyperthermia as monotherapy lasted longer on protein expression than that of the intratumoral 5FU monotherapy (see *y*-axis stating high statistical significance of up-regulated protein expression of C-L and H-L in Supplementary Figure S2).

**Figure 1:**
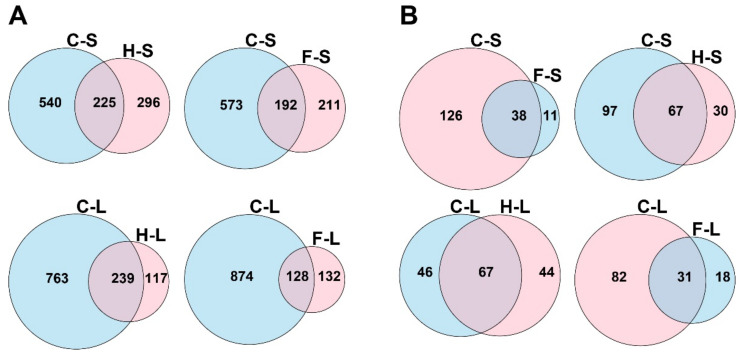
Venn diagrams depicting the differentially expressed proteins (**A**), and significantly enriched canonical pathways (**B**) in different groups compared with the corresponding control. C: combination therapy (thermo-chemotherapy), H: hyperthermia alone, F: 5FU alone, S: short-term, L: long-term.

The Venn diagrams revealed that the highest change of protein expression is to be allocated for the thermo-chemotherapeutic tumor therapy followed by treatment arm “magnetic hyperthermia”. For the treatment of tumors with 5FU alone, the number of differentially expressed proteins was comparatively lower (number of differentially expressed proteins, short- and long-term post observation times, Figure 1A). Next, we had a look on the “nature” of the molecular mechanisms and found that the thermo-chemotherapeutic tumor therapy shared the highest number of significantly enriched canonical pathways with magnetic hyperthermia, and to a lower extent with 5FU, both as monotherapy (number of enriched pathways in Figure 1B).

**Figure 2:**
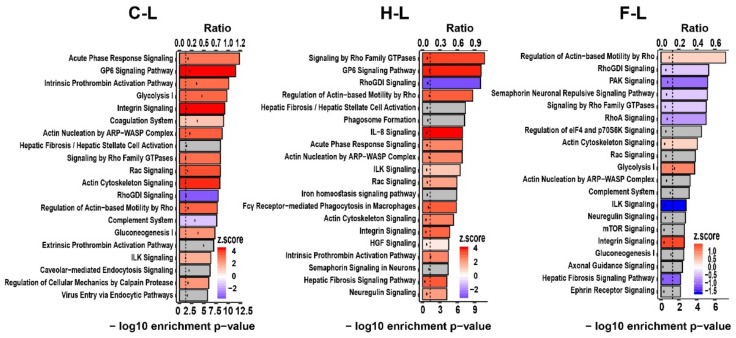
Top 20 significantly enriched canonical pathways of the three experimental tumor therapy arms identified using the IPA software. The length of the bars shows the −log_10_ of the enrichment *p*-value (the lower *x*-axis) and the black dots show the ratio of pathway-related genes differentially expressed in the indicated comparison to the total number of genes in the pathway (the upper *x*-axis). Colors indicate predicted activity *z*-score (positive values (red color) indicate activation, negative values (blue color) indicate inhibition, and gray color indicates pathways for which IPA could not predict the activity). The dashed line shows the significance threshold (log_10_ of 0.05). C: combination therapy (thermo-chemotherapy), H: hyperthermia alone, F: 5FU alone, L: long-term.

In a next step, we identified the most prominent (top 20) and long-lasting canonical pathways. In this context, the thermo-chemotherapeutic tumor treatment shared up-regulated canonical pathways with the magnetic hyperthermia tumor therapy arm, i.e., GP6 signaling pathway, acute phase response signaling, intrinsic prothrombin activation pathway, integrin signaling pathway, glycolysis I, coagulation system, etc. (Figure 2). In contrast, for the intratumoral application of 5FU, mostly down-regulated pathways were predicted among the top-20 identified canonical ones (Figure 2). For all therapeutic arms, some of the mentioned canonical pathways were already conspicuous in the short-post observation time (Supplementary Figure S3).

### 3.4. Functional Allocation of Enriched Canonical Pathways in Treated Tumor Tissues

**Figure 3:**
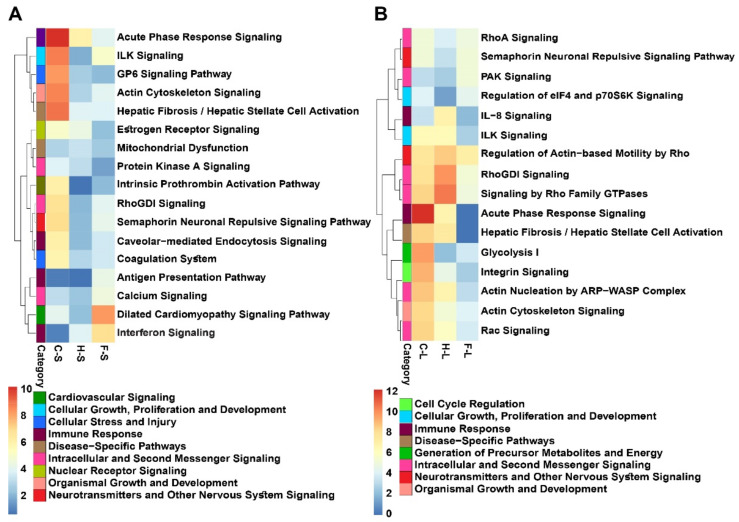
Heatmaps of the top 10 significantly enriched canonical pathways (short-term (**A**) and long-term (**B**) observation periods) of the three experimental tumor therapy arms identified using Table 1. of enrichment *p*-value. C: combination therapy (thermo-chemotherapy), H: hyperthermia alone, F: 5FU alone, S: short-term, L: long-term.

To gain insight into the nature of the most affected molecular pathways amongst the three treatment modalities, we used heat maps of the top 10 significantly enriched canonical protein pathways in each tumor treatment condition (based on the enrichment *p*-value) and allocated them on the basis of their functional categories. In this context, the thermo-chemotherapeutic tumor treatment presented with enriched canonical pathways, related to (a) cellular stress and injury, (b) intracellular and second messenger signaling, (c) nuclear receptor signaling, (d) cellular and organismal growth and development, and (e) immune response shortly after tumor treatment (Figure 3A). In comparison to the combined thermo-chemotherapy approach, magnetic hyperthermia or the 5FU treatment alone showed a comparatively lower effect on the mentioned pathways in the short-time post-observation period (Figure 3B). Interestingly, the impact of the thermo-chemotherapeutic tumor treatment lasted for at least 14 days after the last hyperthermia treatment; several of the mentioned canonical pathways were still enriched. Interestingly, magnetic hyperthermia as monotherapy presented a delayed effect on the mentioned canonical pathways (Figure 3B). Some upregulated canonical pathways were also seen when 5FU only was applied in the tumor (Figure 3B).

#### 3.4.1. Functional Category “Cellular Stress and Injury”

**Figure 4:**
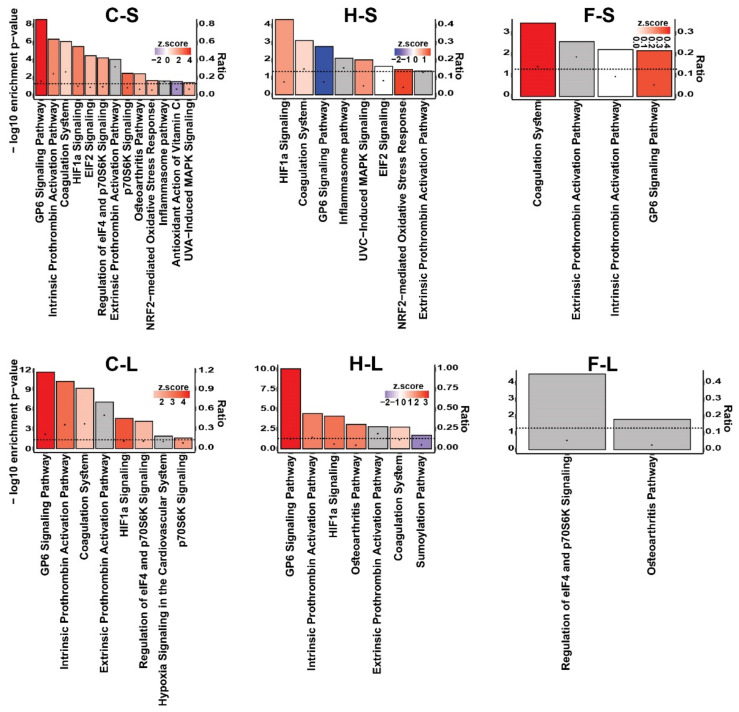
Significantly enriched canonical pathways of the functional category “cellular stress and injury” of the three experimental tumor therapy arms identified by IPA software. The height of the bars shows the −log_10_ of the enrichment *p*-value (right *y*-axis) and the black dots show the ratio of pathway-related genes differentially expressed in the indicated comparison to the total number of genes in the pathway (left *y*-axis). The colors show the predicted activity *z*-score (positive values (red color) indicate activation, negative values (blue color) indicate inhibition, and gray color indicates the pathways whose activity could not be predicted). The dashed line shows the significance threshold (−log_10_ of 0.05). C: combination therapy (thermo-chemotherapy), H: hyperthermia alone, F: 5FU alone, S: short-term, L: long-term.

The functional category “cellular stress and injury” of the thermo-chemotherapeutic tumor therapy modality was due to up-regulated GP6 signaling pathway mentioned above, together with an upregulated (a) intrinsic prothrombin activation pathway, (b) HIF-1a signaling, ribosomal protein S6 kinase (p70s6k) signaling, but also to up-regulated, albeit to a minor extent, coagulation system, EIF2 signaling, etc. In particular, it becomes apparent that such protein pathways were persistently upregulated in the thermo-chemotherapeutic tumor therapy and with some delay in the magnetic hyperthermia therapy arm, whereas such an impact was almost inconspicuous in the intratumoral 5FU one (Figure 4).

#### 3.4.2. Functional Category “Intracellular and Second Messenger Signaling”

**Figure 5:**
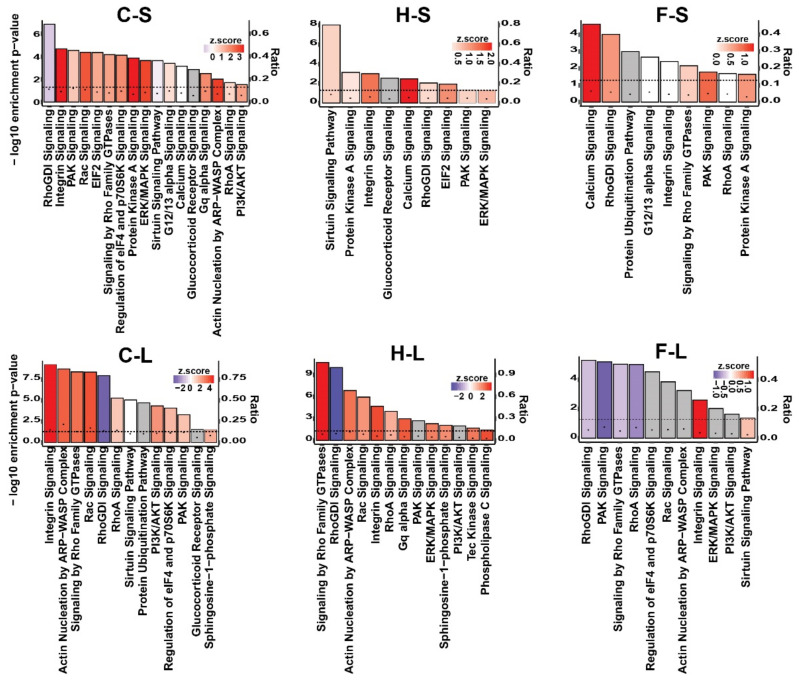
Significantly enriched canonical pathways of the functional category “intracellular and second messenger signaling” of the three experimental tumor therapy arms identified by IPA software. The height of the bars shows the −log_10_ of the enrichment *p*-value (right *y*-axis) and the black circles show the ratio of pathway-related genes differentially expressed in the indicated comparison to the total number of genes in the pathway (left *y*-axis). The colors show the predicted activity *z*-score (positive values (red color) indicate activation, negative values (blue color) indicate inhibition, and gray color indicates the pathways whose activity could not be predicted). The dashed line shows the significance threshold (−log_10_ of 0.05). C: combination therapy (thermo-chemotherapy), H: hyperthermia alone, F: 5FU alone, S: short-term, L: long-term.

With consideration of category named “intracellular and second messenger signaling”, the thermo-chemotherapeutic tumor therapy revealed up-regulated integrin, protein kinase A, ERK/MAPK, Rac, PAK, PI3K/AKT signaling pathways, etc. There was a distinct up-regulation of intracellular and second messenger signaling visible only in relation to the chemotherapy and the magnetic hyperthermia therapy arms, whereas intratumoral 5FU induced rather a down-regulation of Rho and PAK signaling (Figure 5).

#### 3.4.3. Functional Category “Immune Responses”

**Figure 6:**
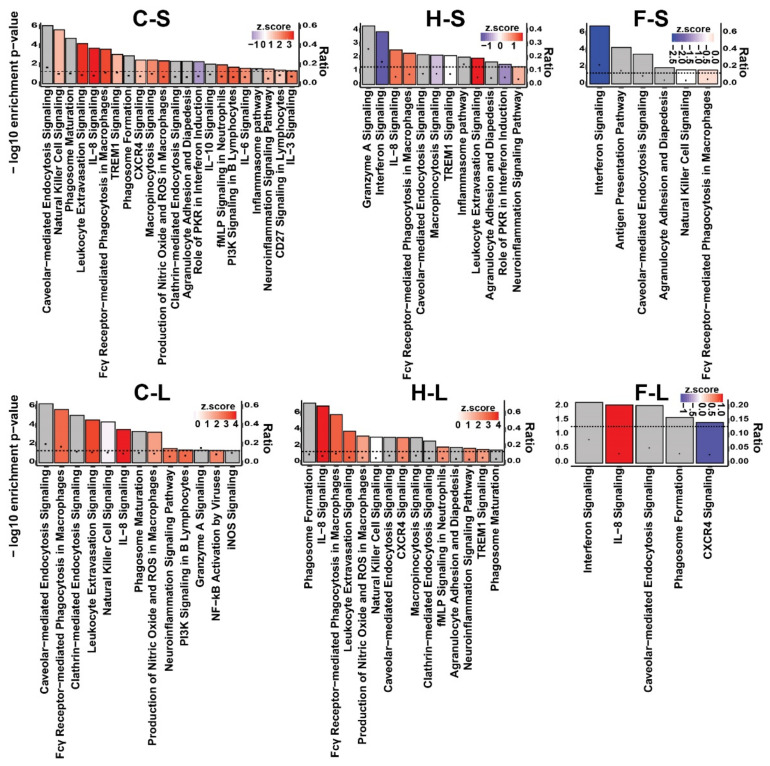
Significantly enriched canonical pathways of the functional category “cellular immune response” of the three experimental tumor therapy arms identified by IPA software. The height of the bars shows the −log_10_ of the enrichment *p*-value (right *y*-axis) and the black circles show the ratio of pathway-related genes differentially expressed in the indicated comparison to the total number of genes in the pathway (left *y*-axis). The colors show the predicted activity *z*-score (positive values (red color) indicate activation, negative values (blue color) indicate inhibition, and gray color indicates the pathways whose activity could not be predicted). The dashed line shows the significance threshold (−log_10_ of 0.05). C: combination therapy (thermo-chemotherapy), H: hyperthermia alone, F: 5FU alone, S: short-term, L: long-term.

With consideration of the category named “immune responses”, the thermo-chemotherapeutic tumor therapy and magnetic hyperthermia alone both induced a persistent up-regulation of leukocyte extravasation signaling, IL-8 Signaling, and Fcα receptor-mediated phagocytosis in macrophages and monocytes, while tumor treatment with 5FU as monotherapy led to down-regulation of pathways involved in cellular immune response (long-term post observation time, Figure 6). Only 5FU led to up-regulation of IL-8 signaling pathway in long-term.

### 3.5. Prediction of Upstream Regulators

**Figure 7:**
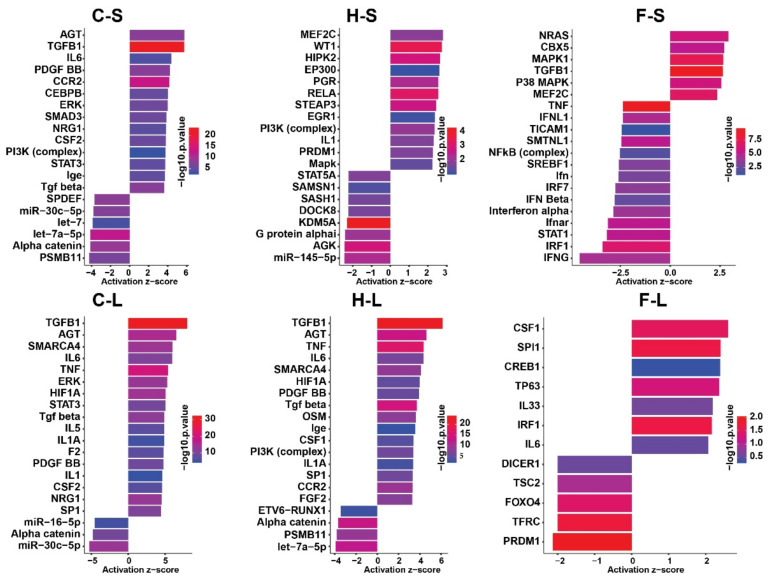
Top 20 predicted upstream regulators of the three experimental therapeutic arms calculated by the IPA software. The length of the bar indicates the activity prediction *z*-score and colors indicate the −log_10_ if enrichment *p*-value. C: combination therapy (thermo-chemotherapy), H: hyperthermia alone, F: 5FU alone, S: short-term, L: long-term.

In order to better understand the reasons why the mentioned were affected by the thermo-chemotherapeutic tumor therapy and of magnetic hyperthermia in particular, we sought to predict the top 20 upstream master regulators (e.g., transcription factors, kinases, etc. based on the activity prediction *z*-score) using IPA software. Hereto, the predicted regulator for the combined thermo-chemotherapeutic tumor therapy were found to belong mostly to the group of cell death/survival associated transcription factors, such as TGF beta, PDGF, ERK, SMAD3, PI3K, STAT3, etc. Further upstream regulators were mainly linked to the inflammatory response and cytokine signaling, including IL-6, CCR2, CEBPB, CSF2, and IgE (Figure 7).

## 4. Discussion

In this study we used computational analysis of the tumor proteome in order to get a comprehensive view on the protein pathways which are responsible for the impact of an iron-oxide nanoparticle and 5-fluorouracil-based nano-hyperthermic approach for the treatment of colon cancers in mice. The thermo-chemotherapeutic tumor treatment has been considerably more effective than magnetic hyperthermia or intratumoral 5FU alone in reducing tumor volumes at comparable therapeutic conditions. Global proteomic analyses revealed 10 pertinent canonical pathways for the thermo-chemotherapeutic tumor treatment, such as GP6 signaling pathway, acute phase response signaling, intrinsic prothrombin activation, integrin signaling, coagulation system, etc. The thermo-chemotherapeutic tumor treatment presented upregulated canonical pathways associated with the functional categories “cellular stress and injury”, “intracellular and second messenger signaling” and “nuclear receptor signaling” “growth proliferation and development” and “immune responses”. Finally, we predict TGF-beta, PDGF, CCR2, TNF, etc., as important upstream regulators in relation to the combined thermo-chemotherapy.

In particular, our data indicate that the distinctly reduced tumor volumes in relation to the combined thermo-chemotherapeutic tumor treatment compared to the monotherapies magnetic hyperthermia and intratumoral 5FU may well be associated with a significantly changed protein expression pattern. Interestingly, the combined thermo-chemotherapeutic tumor treatment shared a high number of canonical pathways particularly with magnetic hyperthermia. This finding elucidates the particular impact of this therapeutic modality in the combined approach. On the other hand, the comparatively low impact of 5FU alone is associated with a drug concentration which corresponds to that of intratumorally injected one, when 5FU was functionalized to the MNP. This was performed to monitor the contribution of the 5FU component of the combined thermo-chemotherapy approach. It is expected that the effects of intratumoral 5FU would be higher in the case of a systemic administration, which would have been associated with distinct side effects for animals (and humans).

The impact of the combined thermo-chemotherapeutic tumor treatment compared to the monotherapies may well be related to the cellular response to stress conditions, since a distinct enrichment of canonical protein pathways was observed in several functional categories. The distinct short-term cellular stress and injury induced by the combined thermo-chemotherapeutic tumor treatment was visible by the enrichment of protein pathways belonging to GP6 signaling pathway and coagulation system, which is in agreement with the high impact of this therapy modality on the tumor volume. GP6 is the major signaling receptor for the extracellular matrix protein, collagen [19], and it plays a critical role in hemostasis and thrombosis through integrin activation, supporting adhesion and the initial stages of platelet aggregation [20,21,22]. In agreement with this, collagen fibers, which are being destroyed by magnetically induced heating, are the most thrombogenic macromolecular components of the extracellular matrix [23]. This means that thrombosis should have greatly contributed to a reduced tumor volume, due to a subsequent block of nutrient supply particularly in tumors treated with the combined thermo-chemotherapy.

Additionally, several pathways associated with functionalities related to intracellular/second messenger and nuclear receptor signaling were found to be enriched, particularly in relation to the combined thermo-chemotherapeutic tumor treatment and with some delay to magnetic hyperthermia as monotherapy. This functional category was identified to encompass enriched canonical pathways associated with the Rho family of GTPases, which suggests cellular response activities against stress and DNA damage [24,25,26]. Further intracellular and second messenger signaling pathways were related to protein kinase A and calcium signaling, albeit they were enriched to a lesser extent. Protein kinase A has shown evidence of serving as a promotor for cell death through phosphorylation of protein targets [27,28,29,30]. Regulative mechanisms for intracellular calcium were also present, albeit with a minor enrichment score. In particular, the accumulation of calcium ions in mitochondria in response to dangerous stimuli leads to increased permeability of mitochondria, release of cytochrome c, and consequently to cell death [31,32]. The distinct tumor volume reduction in response to thermo-chemotherapy may also be related to a transient (short-term) enrichment of estrogen receptor signaling as a further member of functional category “nuclear receptor signaling”. Estrogen receptors are be activated by the presence of absence of estrogen [33,34]. Although activated estrogen receptors involve in signaling pathways that regulate mainly cellular functions, such as growth, differentiation, cell death, and angiogenesis [35], a particular beta class of estrogen receptors is reported to act as an inhibitor of proliferation and growth of colon cancer xenograft [36]. Taken together, the combined thermo-chemotherapy changed intracellular and nuclear receptor signaling which are signs of strong cellular stress.

The enrichment of pathways corresponding to the functional category “cellular and organismal growth and development” was early prominent in relation to the combined thermo-chemotherapeutic tumor treatment, and for magnetic hyperthermia as monotherapy with some delay (long-term, 14 days post therapy). In this context, the ILK signaling pathway was highly enriched, which has been reported to play key roles in cell survival and cell response after stress [37,38,39]. The same applies to actin cytoskeleton signaling, which indicates the attempts of surviving cells to restore intracellular damages [40,41]. This agrees with the observed enrichment of pathways related to the regulation of eIF4 (eukaryotic initiation Factor-4) and p70S6K signaling. Both are known to play critical roles in translational regulation. Hereto, eIF4F is a heterotrimeric protein complex that binds the 5’ cap of messenger RNAs (mRNAs) to promote eukaryotic translation initiation [42] and p70S6K is known to regulate cell growth by inducing protein synthesis components [43].

The combined thermo-chemotherapeutic tumor treatment also seemed to elicit a short-term impact on the immune response, as revealed by the enrichment of pathways belonging to the acute phase response signaling, the caveolar-mediated endocytosis signaling. We postulate that this is mainly related to the clearance of cell debris and denatured proteins and nucleic acids as result of therapy, which is accomplished by immigrated phagocytic cells (e.g., neutrophils, monocytes and macrophages). Such phagocytic cells could well have immigrated into the tumor area after recognition of cellular debris as DAMPs at the tumor site as consequence of the therapy. In agreement with this, a very prominent enrichment was found in relation to the “acute phase response pathway” (APR), particularly after using the combined thermo-chemotherapeutic tumor treatment. This pathway represents a prominent systemic reaction of the organism to local or systemic disturbances in its homeostasis caused by infection, tissue injury, trauma, neoplastic growth or immunological disorders [44]. The antigen presentation pathway was almost inconspicuous as result of the fact that immune-suppressed animals have been used for human xenograft implantation. It is very well conceivable that corresponding T-cell activation processes would have been observed in the case of immunocompetent mice. Additionally, there is increased evidence that coagulation factors are involved in inflammatory responses. In line with this, several of the major coagulation factors, like TF, thrombin, or fibrinogen, are described as potential drivers of inflammation in the bloodstream, but also within tissues [45]. Most of the inflammatory signals which are responsible for immune activation do also precipitate pro-coagulant signals to the coagulation system [46]. In summary, the reduced tumor volumes observed with the combined thermo-chemotherapy are, at least in parts, attributable to immune responses, but more studies with immunocompetent mice are needed.

Despite of the strong anti-tumor effects of the combined thermo-chemotherapeutic tumor therapy, several surviving cells were still able to activate pathways which regulate metabolism, growth, survival of both neoplastic and immune cells, such as IL-8, ERK/MAPK, PI3K/AKT, etc. [47,48,49,50]. In agreement to these findings are the predicted top 20 upstream regulators for the combined thermo-chemotherapeutic tumor therapy. One typical example is TGF-beta, whose signaling pathway has been considered in immune responses but also as tumor suppressor pathway and a promoter of tumor progression and invasion [51]. The same applies for PDGF, ERK, SMAD3, PI3K, and STAT3, which are mainly associated with modulation cellular growth [52,53,54,55]. Further upstream regulators are mainly linked to the inflammatory response and cytokine signaling, such as CCR2, TNF, etc. Such findings indicate the necessity to conduct more than two therapeutic cycles with the combined therapy in order to also kill surviving tumor cells.

By taking the observations of this study together, the reduction of tumor volumes after the administration of a thermo-chemotherapeutic therapy is not only because of tumor cell death and damaged vasculature, but also to alterations of extracellular matrix proteins, i.e. the induction of thrombogenic collagen fibers. Beyond the previously reported DNA damage and increased stress levels in surviving tumor cells after using themo-combinatorial therapies [8,10,56], we showed that there is also a distinct impact on second messenger and nuclear receptor signaling. The fact that the combined thermo-chemotherapy shared most of the pathways with magnetic hyperthermia illustrates its prominent impact and the synergistically increased effects in presence of very low concentrations of 5FU.

## 5. Conclusions

In conclusion, we identified several protein pathway regulators which were responsible for the therapeutic impact of the combined thermo-chemotherapy on the basis of 5FU-MNPs. Namely, such effects are not only due to damaged vasculature as reported before, but also to alterations of extracellular matrix proteins, i.e., the induction of thrombogenic collagen fibers, which ultimately block further nutrient supply to the tumor. Beyond the extensive DNA damage and increased stress levels in surviving cells as published earlier, there is also a distinct impact on second messenger and nuclear receptor signaling. We further associate the clear signs of acute phase response signaling and the caveolar-mediated endocytosis signaling with the recognition of DAMPs at the tumor site by phagocytic cells which immigrated into the tumor area. Finally, some surviving tumor cells may well be responsible for the activation of pathways associated with growth of neoplastic and immune cells, and this fact implies the necessity to conduct multiple therapy cycles in the future, which could be performed in connection with a corresponding marker-based monitoring and the aid of the regulators predicted in this study.

## Figures and Tables

**Table 1:** Relative tumor volumes after thermo-chemotherapy treatment of human HT29 colon tumors compared to hyperthermia and intratumoral 5FU treatments alone and to untreated controls at 14 days after the last magnetic hyperthermia treatment. Tumor volume in percent was evaluated as relative to tumor volumes before starting with the experiments. Mean ± standard deviation; significantly different to non-treated controls with *p* ≤ 0.05 (*) or *p* ≤ 0.01 (**).

	Thermo- Chemotherapy (C)	Hyperthermia Alone (H)	5FU Alone (Intratumoral) (F)	Non- Treated (N)
Tumor volume (%)	159 ± 153 **	494 ± 111 *	757 ± 216	888 ± 189
Animal number	11	11	10	11

## Data Availability

The mass spectrometry proteomics data have been deposited to the ProteomeXchange Consortium (http://proteomecentral.proteomexchange.org) via the PRIDE [57] partner repository with the dataset identifier PXD027656 on date 30 July 2021.

## Supplementary Materials

The following are available online at https://www.mdpi.com/article/10.3390/pharmaceutics13101625/s1, Figure S1: Principal component analysis (PCA) showing clusters of samples based on their similarity, Figure S2: Volcano plots displaying the log_2_ fold change of expression (*x*-axis) and −log_10_ of *q*-value (*y*-axis) for each comparisons, Figure S3: Top 20 significantly enriched canonical pathways of the three experimental tumor therapy arms shortly after therapy as identified using the IPA software.
