# Supplementary material for: Iron Oxide Nanoparticles Carrying 5-Fluorouracil in Combination with Magnetic Hyperthermia Induce Thrombogenic Collagen Fibers, Cellular Stress, and Immune Responses in Heterotopic Human Colon Cancer in Mice
Source: Pharmaceutics. 2021 Oct 6;13(10):1625. doi: 10.3390/pharmaceutics13101625 (PMC8541380; doi:10.3390/pharmaceutics13101625)
Supplement: Supplementary file 1 [file pharmaceutics-13-01625-s001.zip › pharmaceutics-1360476-supplementary.pdf]

pharmaceutics

MDPI

Supplementary Materials: Iron Oxide Nanoparticles Carrying 5-Fluorouracil in Combination with Magnetic Hyperthermia Induce Thrombogenic Collagen Fibers, Cellular Stress, and Immune Responses in Heterotopic Human Colon Cancer in Mice

Mohammad Dabaghi, Seyed Mohammad Mahdi Rasa, Emilio Cirri, Alessandro Ori, Francesco Neri, Rainer Quaas, and Ingrid Hilger

Figures

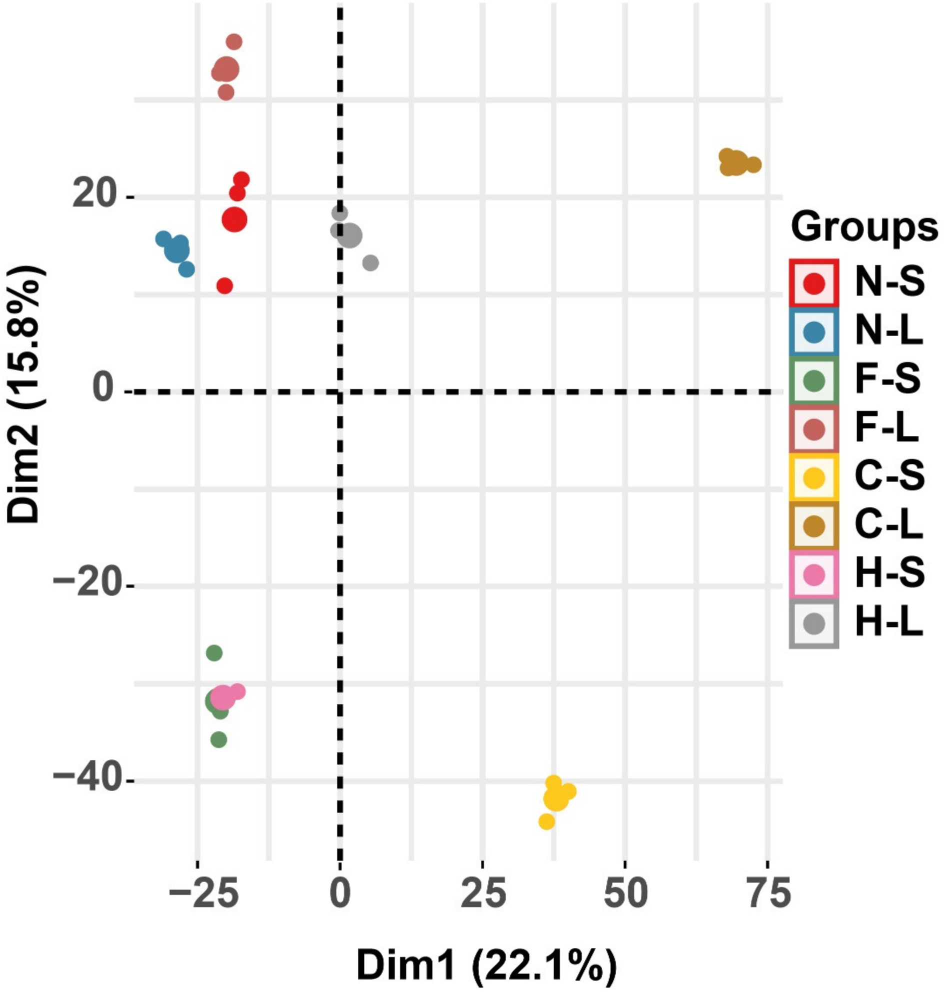

Figure S1. Principal component analysis (PCA) showing clusters of samples based on their similarity. PC1 (Dim1) shows 22.1% and PC2 (Dim2) shows 15.8% of the total variance. The mean of each group is plotted with a larger circle. C: combination therapy (thermo-chemotherapy), H: hyperthermia alone, F: 5FU alone, N: non-treated, S: short-term, L: long-term

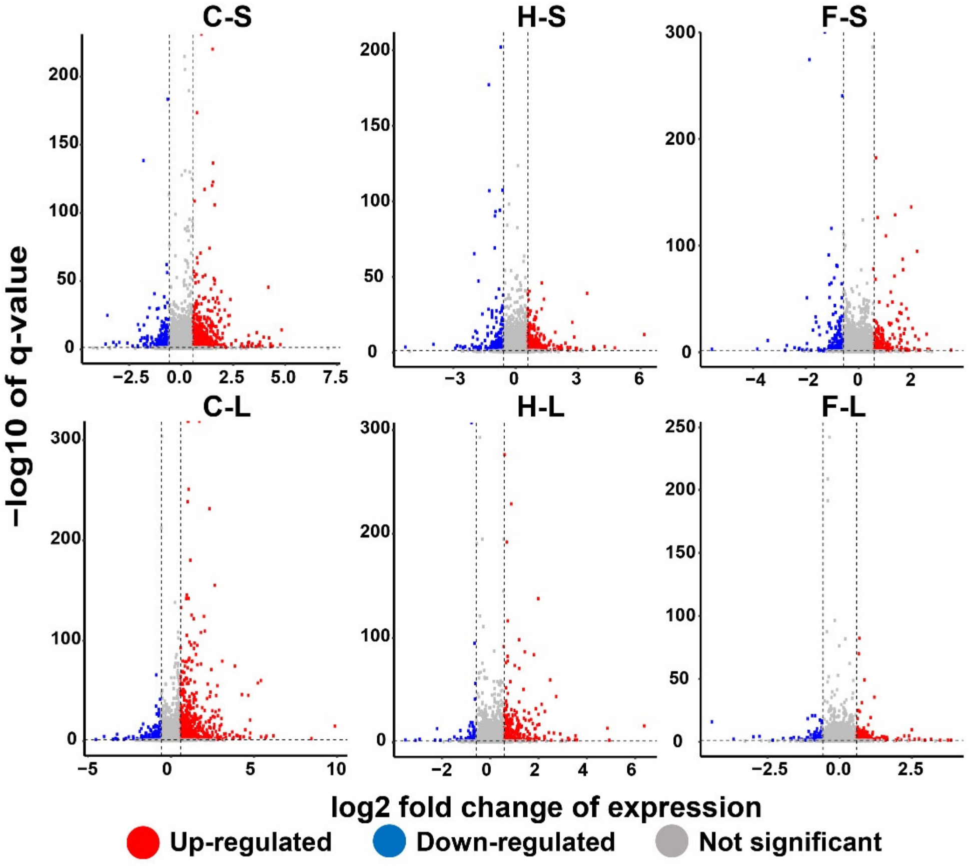

Figure S2. Volcano plots displaying the log2 fold change of expression (x axis) and –log 10 of q-value (y axis) for each comparisons. C: combination therapy (thermo-chemotherapy), H: hyperthermia alone, F: 5FU alone, S: short-term, L: long-term.

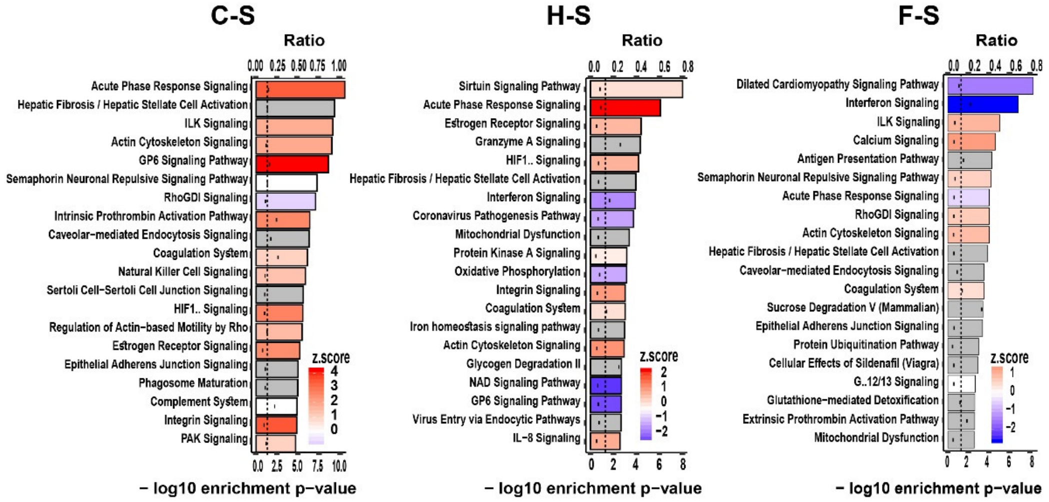

Figure S3. Top 20 significantly enriched canonical pathways of the three experimental tumor therapy arms shortly after therapy as identified using the IPA software. The length of the bars shows the -log 10 of the enrichment p-value (the lower x-axis) and the black dots show the ratio of pathway-related genes differentially expressed in the indicated comparison to the total number of genes in the pathway (the upper x-axis). Colors indicate predicted activity z-score (positive values (red color) indicate activation, negative values (blue color) indicate inhibition, and gray color indicates pathways for which IPA could not predict the activity). The dashed line shows the significance threshold (-log10 of 0.05). C: combination therapy (thermo-chemotherapy), H: hyperthermia alone, F: 5FU alone, S: short-term, L: long-term.
